# Evaluating the performance of a risk assessment score tool to predict HIV acquisition among pregnant and postpartum women in Kenya

**DOI:** 10.1371/journal.pone.0306992

**Published:** 2024-07-10

**Authors:** Nok Chhun, Claire W. Rothschild, Monalisa Penumetsa, Daniel Matemo, Peninah Kithao, Barbra A. Richardson, Grace John-Stewart, John Kinuthia, Alison L. Drake

**Affiliations:** 1 Department of Global Health, University of Washington, Seattle, WA, United States of America; 2 Department of Epidemiology, University of Washington, Seattle, WA, United States of America; 3 Research and Programs, Kenyatta National Hospital, Nairobi, Kenya; 4 Department of Biostatistics, University of Washington, Seattle, WA, United States of America; 5 Department of Medicine, University of Washington, Seattle, WA, United States of America; 6 Department of Pediatrics, University of Washington, Seattle, WA, United States of America; International AIDS Vaccine Initiative, UNITED STATES OF AMERICA

## Abstract

**Background:**

Clinical risk score tools require validation in diverse settings and populations before they are widely implemented. We aimed to externally validate an HIV risk assessment tool for predicting HIV acquisition among pregnant and postpartum women. In the context of prevention of mother-to-child transmission programs, risk score tools could be used to prioritize retesting efforts and delivery of pre-exposure prophylaxis (PrEP) to pregnant and postpartum women most at risk for HIV acquisition while minimizing unnecessary perinatal exposure.

**Methods:**

Data from women enrolled in a cross-sectional study of programmatic HIV retesting and/or receiving maternal and child health care services at five facilities in Western Kenya were used to validate the predictive ability of a simplified risk score previously developed for pregnant/postpartum women. Incident HIV infections were defined as new HIV diagnoses following confirmed negative or unknown status during pregnancy. Predictive performance was assessed using the area under the receiver operating characteristic curve (AUC) and Brier score.

**Results:**

Among 1266 women with 35 incident HIV infections, we found an AUC for predicting HIV acquisition of 0.60 (95% CI, 0.51, 0.69), with a Brier score of 0.27. A risk score >6 was associated with a 2.9-fold increase in the odds of HIV acquisition (95% CI, 1.48, 5.70; p = 0.002) vs scores ≤6. Women with risk scores >6 were 27% (346/1266) of the population but accounted for 52% of HIV acquisitions. Syphilis, age at sexual debut, and unknown partner HIV status were significantly associated with increased risk of HIV in this cohort.

**Conclusion:**

The simplified risk score performed moderately at predicting risk of HIV acquisition in this population of pregnant and postpartum women and may be useful to guide PrEP use or counseling.

## Introduction

Clinical risk scores have been utilized in a variety of settings and populations to identify individuals who are at highest risk for HIV acquisition [1–5]. A prior risk score developed specifically for pregnant and postpartum women was moderately predictive of new HIV acquisition (area under the curve [AUC] of 0.76 (95% Confidence Interval [CI], 0.67, 0.85)) in Kenya [6]. However, this tool has not been externally validated. The tool was developed prior to the introduction of pre-exposure prophylaxis (PrEP) for pregnant and postpartum women. In resource-limited settings, a risk score could be used to prioritize HIV retesting efforts in prevention of mother-to-child transmission (PMTCT) programs, and delivery of PrEP to pregnant and breastfeeding women most at risk for HIV acquisition while minimizing unnecessary perinatal exposure.

Strategies that identify and prevent incident maternal HIV acquisition during pregnancy and the postpartum period are needed for elimination of mother-to-child transmission (EMTCT) [7–9]. While maternal HIV incidence is high in sub-Saharan Africa, recent trends suggest incidence is declining from 3.8 per 100 person-years (PY) between 1980–2012 [10] to 2.1 per 100 PY after 2014 [11]. One strategy for detecting incident maternal HIV, as recommended by the World Health Organization (WHO), is maternal HIV retesting in the third trimester, with catch-up testing at labor, delivery, and early postpartum [12]. Some countries may also consider additional time points later in the postpartum period to retest women [12]. This approach to retesting promotes detection of new maternal HIV infections among women who may not have tested during antenatal care or who initially tested negative and acquired HIV later in their pregnancy or during the postpartum period.

Current WHO guidelines recommend PrEP as an HIV prevention strategy for pregnant and breastfeeding women in high incidence settings [12,13]. Increased engagement with the healthcare system as a result of retesting efforts offers healthcare providers an opportunity to screen and counsel women at high risk for HIV acquisition about initiating PrEP. Prioritizing PrEP provision to women at highest need may reduce HIV transmission from their sexual partners and reduce perinatal transmission. Use of HIV risk assessment tools in antenatal and postnatal care settings may have potential to prevent and identify incident maternal HIV acquisition, minimizing additional resources necessary under a universal screening approach. Targeting resources to women at highest risk while limiting potential perinatal exposure is an approach that optimizes PrEP use during pregnancy and postpartum [14–16]. As such, efforts to evaluate how a risk score tailored for peripartum women performs in the context of declining incidence and roll-out of PrEP in pregnancy may be helpful in assessing utility of this approach.

We externally validated an existing HIV risk score designed for pregnant and postpartum women in a new population of pregnant and postpartum women from the same region in Kenya [17]. We also examined whether there were additional predictors of HIV acquisition that have emerged since development of the initial risk score.

## Materials and methods

### Study population

Data were from a cross-sectional study on maternal HIV retesting and a retrospective review of programmatic HIV retesting documented in maternal child health booklets; both studies aimed to identify incident HIV infections among Kenyan women receiving maternal and child health care services as previously described [18]. In the cross-sectional study, participants were enrolled between January 2017 and July 2019 from two health facilities, Ahero sub-District and Bondo District Hospitals in western Kenya. Women were enrolled during the third trimester of pregnancy (≥28 weeks gestation), at delivery, or at 6 weeks, 6 months, or 9 months postpartum. Women were eligible for study participation if they were ≥14 years; tested HIV-negative ≥3 months prior (if enrolled during pregnancy, labor and delivery, or 6 weeks postpartum), had no documentation of an initial test, or had an unknown HIV status (if enrolled during the labor and delivery or the postpartum period); and were willing to provide written informed consent. At enrollment, all women were administered a survey on sociodemographic, partner characteristics, sexual risks and behaviors, reproductive, clinical and HIV history information. Syphilis serology results were abstracted from maternal child health (MCH) booklets. After survey administration, HIV testing and counseling was conducted per Government of Kenya guidelines [19]; women newly diagnosed with HIV were referred for follow-up care and treatment at MCH clinics [18].

Women in the retrospective chart review of programmatic HIV retesting study had data abstracted when they were enrolled at 6 weeks or 9 months postpartum from January to July 2019, also from Ahero sub-District and Bondo District, in addition to three other health facilities, Rachuonyo and Siaya District Hospitals in Western Kenya, and Riruta Health Centre in Nairobi. Women identified with incident HIV were administered the same sociodemographic and behavioral survey as participants enrolled in the cross-sectional study, as previously described [18]. Similarly, syphilis serology was also abstracted from MCH booklets.

Incident HIV infections were defined as those with a documented prior negative HIV infection during or after pregnancy or unknown status during pregnancy. In addition, active case finding for pregnant and postpartum women with prior known incident maternal infection (negative rapid HIV test followed by later positive test before 1 year postpartum) reported by healthcare providers at the study sites was conducted; HIV retesting information was abstracted from programmatic MCH data at the current visit or retrospectively for women enrolled from both studies. In summary, the study population included women with HIV who were identified from the cross-sectional study, case finding, and from the programmatic HIV retesting data abstraction study, and HIV-negative women from the cross-sectional study. HIV-negative women who were enrolled in the programmatic HIV retesting data abstraction study were excluded because HIV risk factors were not collected.

Study procedures were approved by the Kenyatta National Hospital/University of Nairobi Ethics and Research Committee and the University of Washington Institutional Review Board. All authors had access to information that could identify individual participants during data collection; however, data were de-identified for analysis and accessed from May 2020 to May 2023. All participants provided written informed consent.

### Statistical analysis

We aimed to externally validate the previously developed risk score [6]. The analytic sample was comprised of women who had complete data on all risk factors included in the simplified version of the risk score described by Pintye et al which focused on indicators routinely assessed in MCH settings; these include total number of lifetime sexual partners, male partner with unknown HIV status, and reactive syphilis serology test in pregnancy via rapid plasma reagin (RPR). The risk score was derived by assigning the following point values each indicator: one per sexual partner, six for a partner with unknown status, five for a reactive RPR, and zero if for none of these indicators. Since laboratory test results for bacterial vaginosis and candidiasis were not conducted as part of the cross-sectional study, nor routinely performed and documented in medical records, it was not possible to externally validate the full risk score.

In the external validation of the simplified risk score, individual risk score values were calculated for each woman. A score cut-point of 6 was used to define high risk corresponding to the previously developed score. Predictive ability of the simplified risk score, and the multivariable model from which the score was derived [6], was evaluated through estimation of area under the receiver operating characteristic curve (AUC-ROC). Further validation of the predictive ability of the risk score was done using 10-fold cross validation, a method which randomly partitions the data into 10 random subsets to calculate an average AUC [20]. The average AUC from the 10-fold cross validation was compared to the AUC from the complete dataset to ascertain robustness of the risk score tool using AUC values <0.7 to reflect poor performance [21]. Sensitivity, specificity, positive predictive value (PPV), and negative predictive value (NPV) were estimated with varying risk score cut-points; with an optimal cut-point defined by Youden’s J statistic. Additionally, the number needed to screen (NNS) at each cut-point to detect one individual with HIV was calculated using the inverse of the predictive summary index (PSI), 1/PSI. The PSI is determined from the formula PPV + NPV– 1 [22]. The Brier score was also calculated to assess overall model performance, using a threshold of <0.25, which indicates the risk score is informative for prediction [20].

After external validation of the risk score, we examined whether additional predictors of HIV acquisition emerged since development of the initial risk score. We assessed this by constructing logistic regression models to characterize the relationship between each independent variable included in the initial risk score developed by Pintye et al., as well as additional correlates not assessed during initial risk score development (familiarity with PrEP as an HIV prevention intervention, and ever use of PrEP) [6]. Variables associated with HIV acquisition at p<0.10 in the univariate models were included in a multivariable model. If potential predictors were collinear, the variable with greater clinical relevance based on the literature was chosen for inclusion. The analysis was restricted to women with RPR information. We also conducted an exploratory analysis where if collinearity was detected, variables with the least missing data were chosen for inclusion in the multivariable model (S1 Table). Covariates included in the multivariable model were then assessed in a stepwise logistic regression model based on the minimum Akaike information criterion (AIC) [23] to identify the combination of factors that best predicted HIV acquisition.

A sensitivity analysis was conducted excluding women enrolled in other studies on maternal PrEP use as they may have higher risks of HIV acquisition and their inclusion may bias the risk score towards higher predictive ability of HIV acquisition. All analyses were conducted in Stata version 16.0 (StataCorp, College Station, TX), with cross validation analysis using the cvauroc package, and model selection using stepwise logistic regression with estat ic command post estimation.

## Results

In total, 2770 women were identified in the cross-sectional study (2761 HIV-negative, 9 incident HIV infections) and an additional 36 incident infections (27 identified through active case finding, 9 through the programmatic HIV retesting data abstraction study). We included 1266 (45%) of these women in this analysis (1231 HIV-negative, 35 incident infections); women were excluded from the analysis if they had missing data on syphilis RPR (n = 1424), lifetime number of sexual partners (n = 107), and/or missing partner HIV status (n = 9).

Among all women, the overall median age was 23 years (interquartile range [IQR]: 20, 27), the majority (62%) of whom were between 21–30 years (Table 1). Most (72%) were currently in a relationship, for a median duration of 4 years, and with an older male partner (median 5 years older, IQR: 3, 7). One-third had a male partner with unknown HIV status. The median number of lifetime sexual partners was 2 (IQR: 1, 3). The majority (66%) reported having ever heard of PrEP, and among those who had heard of PrEP, only 8% had ever used PrEP. None of the women had an unknown HIV status at the time of enrollment. Among the 35 women with incident HIV infections included in the risk score validation, 15 (43%) were detected during pregnancy (9 during the third trimester, 6 at delivery) and 20 (57%) in the postpartum period (9 at 6 weeks, 2 at 6 months, and 9 at 9 months).

**Table 1:** Baseline characteristics of women receiving maternal and child health care during pregnancy and postpartum in western Kenya.

	All women (n = 2806)		HIV-seronegative (n = 2761)		HIV-seropositive (n = 45)		Risk Score Dataset (n = 1266)		Risk Score HIV-seronegative (n = 1231)		Risk Score HIV-seropositive (n = 35)	
	N	Median (IQR) or n (%)	N	Median (IQR) or n (%)	N	Median (IQR) or n (%)	N	Median (IQR) or n (%)	N	Median (IQR) or n (%)	N	Median (IQR) or n (%)
**Demographic characteristics**												
Age (years)*	2806	23 (20–27)	2761	23 (20–27)	45	23 (20–28)	1266	23 (20–28)	1231	23 (20–27)	35	23 (20–29)
Age category (years)												
<21		781 (28)		769 (28)		12 (27)		345 (27)		334 (27)		11 (31)
21–30		1748 (62)		1720 (62)		28 (62)		777 (61)		758 (62)		19 (54)
>30		277 (10)		272 (10)		5 (11)		144 (11)		139 (11)		5 (14)
Education												
Completed education (years)	2802	12 (8–13)	2757	12 (8–13)	45	10 (8–12)	1264	12 (8–13)	1229	12 (8–13)	35	10 (8–12)
Completed secondary education	2805	1455 (52)	2760	1440 (52)	45	15 (33)	1266	660 (52)	1231	647 (53)	35	13 (37)
Completed education <8 years	2802	238 (8)	2757	235 (9)	45	3 (7)	1264	106 (8)	1229	103 (8)	35	3 (9)
Neither parent alive	2804	365 (13)	2759	357 (13)	45	8 (18)	1265	200 (16)	1230	192 (16)	35	8 (23)
Monthly household income ≥10,000KSH	1784	703 (39)	1759	693 (39)	25	10 (40)	728	354 (49)	711	345 (49)	17	9 (53)
**Relationship history**												
Marital status (married)	2765	1868 (68)	2720	1832 (67)	45	36 (80)	1255	931 (74)	1220	904 (74)	35	27 (77)
Has current partner	2797	2010 (72)	2752	1973 (72)	45	37 (82)	1266	985 (78)	1231	958 (78)	35	27 (77)
Relationship duration (years)^1^	2000	4 (2–7)	1964	4 (2–7)	36	3 (2–7)	981	4 (2–8)	955	4 (2–8)	26	4 (2–10)
Polygamous relationship	1990	153 (8)	1953	146 (7)	37	7 (19)	975	60 (6)	948	54 (6)	27	6 (22)
**Partner characteristics**												
Partner age difference (years older)	1883	5 (3–7)	1849	5 (3–7)	34	6 (4–8)	915	5 (3–7)	889	5 (3–7)	26	7 (5–9)
Partner completed secondary education	1962	1380 (70)	1927	1362 (71)	35	18 (51)	960	692 (72)	934	677 (72)	26	15 (58)
Partner uncircumcised	1945	584 (30)	1910	578 (30)	35	6 (17)	955	301 (32)	929	297 (32)	26	4 (15)
Partner HIV status^1^	1998		1961		37		985		958		27	
Negative		1319 (66)		1306 (67)		13 (35)		657 (67)		646 (67)		11 (41)
Positive		21 (1)		15 (<1)		6 (16)		9 (<1)		4 (<1)		5 (19)
Unknown		658 (33)		640 (33)		18 (49)		319 (32)		308 (32)		11 (41)
Partner currently on ART^2^	21	17 (81)	15	12 (80)	6	5 (83)	9	8 (89)	4	3 (75)	5	5 (100)
**Sexual behavior and characteristics**												
Age at sexual debut (years)^3^	2516	16 (15–18)	2476	16 (15–18)	40	15 (15–17)	1151	17 (15–18)	1119	17 (15–18)	32	15 (14–17)
Lifetime number of sexual partners	2686	2 (1–3)	2643	2 (1–3)	43	2 (1–2)	1266	2 (1–3)	1231	2 (1–3)	35	2 (1–2)
Any condomless sex in the last month	980	870 (89)	966	861 (89)	14	9 (64)	413	376 (91)	401	368 (92)	12	8 (67)
Frequency of condomless sex in the last month	980	3 (2–5)	966	3 (2–5)	14	4 (0–6)	413	3 (2–5)	401	3 (2–5)	12	4 (0–7)
**STIs and genital tract infections**												
History STIs	2795	39 (1)	2750	36 (1)	45	3 (7)	1260	24 (2)	1225	21 (2)	35	3 (9)
Ever diagnosed with syphilis^4^	1298	10 (<1)	1268	8 (<1)	30	2 (7)	1194	10 (<1)	1166	8 (<1)	28	2 (7)
**Knowledge of PrEP**												
Ever heard of PrEP	1381	914 (66)	1344	891 (66)	37	23 (62)	1265	862 (68)	1230	840 (68)	35	22 (63)
Ever used PrEP	914	75 (8)	891	72 (8)	23	3 (13)	862	69 (8)	840	66 (8)	22	3 (14)

*Eligibility criteria for participation is maternal age at least 14 years old; although missing in database for some women, age criteria was verbally confirmed prior to study enrollment.

^1^Among women with current partners

^2^Among women with current partners with HIV

^3^Excluded women who did not know

^4^Syphilis results not missing at random but was not captured prior to March 2018. Abbreviations: ART, antiretroviral therapy; IQR, interquartile range; STI, sexually transmitted infection; PrEP, pre-exposure prophylaxis.

**Fig 1:**
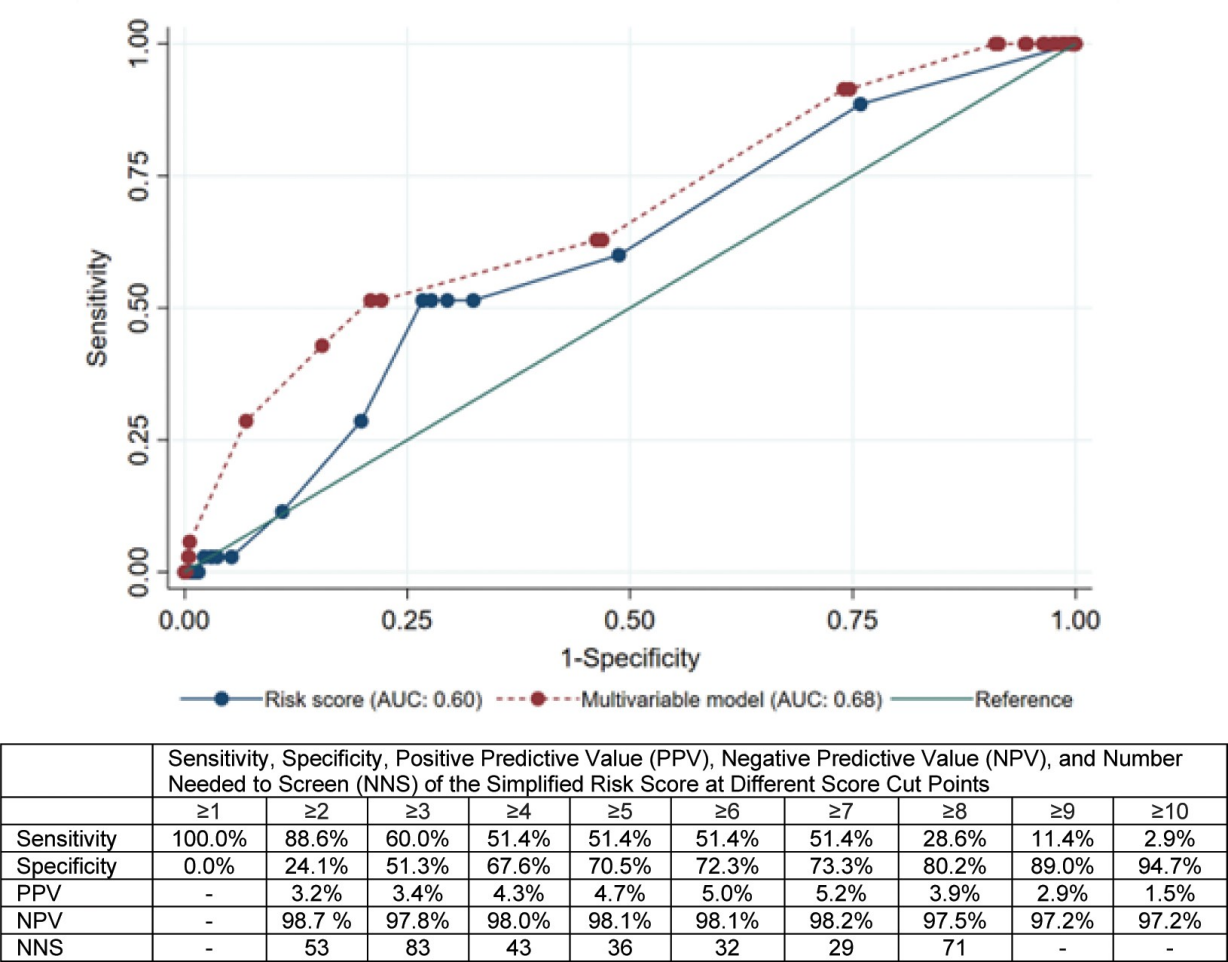
Receiver operating characteristic curve (ROC) analysis of continuous simplified risk score and multivariable model. Notes: Risk score and multivariable logistic model both include number of sexual partners, knowledge of partners’ HIV status, and syphilis status.

In the cohort, the median simplified risk score was 2 (IQR: 2, 7), with 27% (347/1266) defined as high-risk among all women, and 51% of women with incident HIV (18/35) identified as high risk (Table 2). A risk score >6 was associated with a 2.9-fold increase in the odds of HIV acquisition (95% CI, 1.48, 5.70; p = 0.002) and corresponded to an AUC for predicting HIV acquisition of 0.60 (95% CI, 0.51, 0.69; Table 3 and Fig 1), with a Brier score of 0.27. For each additional point increase in the risk score there was a trend for increasing odds of HIV acquisition (OR = 1.06, 95% CI, 0.99, 1.13; p = 0.09). Results were similar in a sensitivity analysis excluding 93 women (n = 1 with incident maternal HIV) co-enrolled in PrEP studies from the analysis.

**Table 2:** Descriptive statistics of simplified risk score and odds of HIV seroconversion associated with risk score value.

	External Validation Dataset (N = 1266)			Excluding women enrolled PrEP studies (N = 1173)		
	Median (IQR) or n (%)	OR (95% CI)	p-value	Median (IQR) or n (%)	OR (95% CI)	p-value
Continuous score	2 (2, 7)	1.06 (0.99, 1.13)	0.09	2 (2, 7)	1.08 (0.99, 1.17)	0.07
Score >6	347 (27)	2.90 (1.48, 5.70)	0.002	320 (27)	2.76 (1.39, 5.47)	0.004

**Table 3:** Area under the receiver operating characteristic curve (AUC-ROC) analysis.

	External Validation Dataset	Excluding women enrolled in PrEP studies
	AUC-ROC (95% CI)	AUC-ROC (95% CI)
Continuous score	0.60 (0.51, 0.69)	0.60 (0.51, 0.69)
Multivariable model	0.68 (0.58, 0.77)	0.66 (0.57, 0.76)
Brier score (>6 points)	0.27	0.27

**Fig 2:**
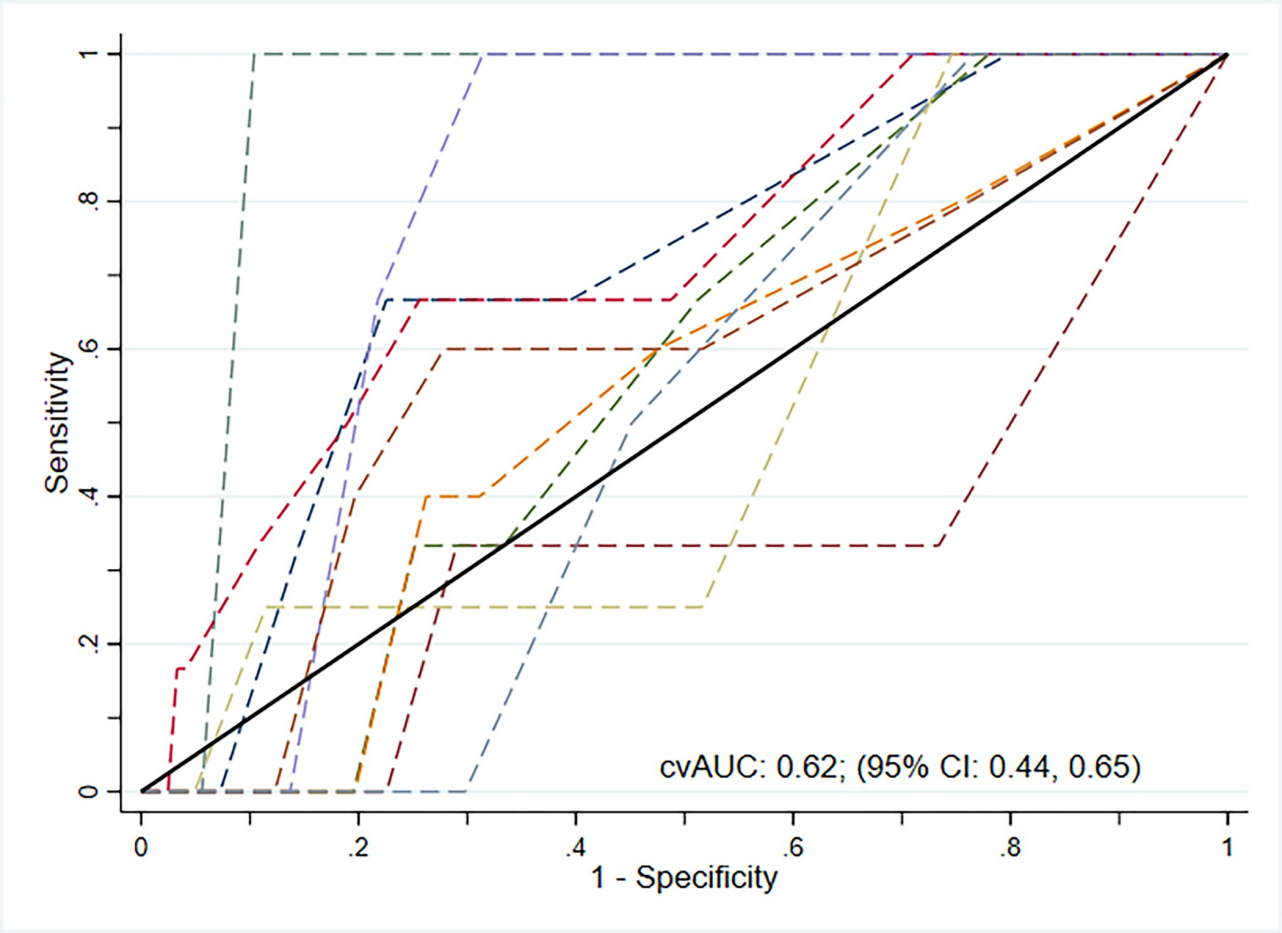
10-fold cross-validated AUC-ROC. Notes: 95% confidence intervals (CI) calculated using the bootstrap bias-corrected approach. Cross-validation conducted using Stata package *cvauroc*. Each dashed line represented one of the 10-fold samples; cvAUC = cross-validated area-under-the-receiver-operating-characteristic-curve.

Using the external validation sample, the risk score cut-point used was 6, with 51.4% sensitivity and 72.3% specificity, 5.0% PPV, and 98.1% NPV. The number needed to screen to identify one individual with HIV was 32. In the cross-validation of the continuous risk score, the average AUC was 0.62 (95% CI, 0.44, 0.65; Fig 2).

In univariate logistic regression models, risk of HIV acquisition was associated with syphilis infection, a history of STIs, having a polygamous relationship, age at sexual debut less than 17 years, having a male partner of unknown HIV status, and having an older partner (Table 4). Differences in HIV risk by PrEP knowledge or ever use of PrEP were not detected. Having syphilis, an age of sexual debut less than 17 years, and having a male partner of unknown HIV status (compared to those with a negative or positive status) remained significantly associated with HIV acquisition in the multivariable model. In the stepwise model selection, syphilis infection (OR = 7.48, 95% CI: 1.48, 37.83), having an age at sexual debut less than 17 years (OR = 2.87, 95% CI: 1.27, 6.49), and having a male partner of unknown HIV status (OR = 2.53, 95% CI: 1.17, 5.49) were associated with maternal HIV acquisition.

**Table 4:** Correlates of HIV acquisition.

	Univariate^a^			Multivariable^b^		Stepwise^c^	
Enrollment characteristics	n^a^	OR (95% CI)	p-value	aOR (95% CI)	p-value		aOR (95% CI)	p-value
Demographic characteristics							
Age <21 y	1382	1.19 (0.58, 2.43)	0.64				
Education <8 y	1380	0.99 (0.30, 3.27)	0.99				
Married	1370	1.23 (0.56, 2.72)	0.61				
Polygamous relationship*	1069	5.20 (2.14, 12.65)	<0.001				
Relationship duration (in years)*	1076	1.03 (0.95, 1.11)	0.47				
Partner characteristics							
Partner age difference (years older)*	1002	1.10 (1.03, 1.18)	0.01				
Partner uncircumcised*	1050	0.36 (0.12, 1.04)	0.06				
Partner HIV status unknown	1372	2.96 (1.38, 6.33)	0.01	2.53 (1.17, 5.49)	0.01	2.53 (1.17, 5.49)	0.02
No partner	1379	1.00 (0.45, 2.21)	1.00	1.56 (0.62, 3.94)	0.35	1.56 (0.62, 3.94)	0.35
Sexual behavior and practices							
Age at sexual debut <17 y	1382	3.15 (1.40, 7.07)	0.01	2.87 (1.27, 6.49)	0.01	2.87 (1.27, 6.49)	0.01
Age at sexual debut unknown	1382	0.91 (0.35, 2.37)	0.85	1.63 (0.52, 5.11)	0.40	1.63 (0.52, 5.11)	0.41
Lifetime no. of sexual partners^§^	1382	0.85 (0.42, 1.73)	0.66				
Any condomless sex in the past month	1382	1.01 (0.90, 1.14)	0.83				
Ever heard of PrEP	1381	0.84 (0.43, 1.64)	0.60				
Ever used PrEP	1381	1.56 (0.47, 5.20)	0.47				
STI and genital tract infections							
History of STIs	1376	5.28 (1.51, 18.50)	0.01				
Syphilis	1382	9.55 (1.96, 46.62)	0.01	7.48 (1.48, 37.83)	0.02	7.48 (1.48, 37.83)	0.02

Notes

* among partnered women only in unadjusted analyses; polygamous relationship excluded to allow unmarried women to be included in the multivariable model

^§^ Dichotomized at 2 or more sexual partners

^a^ Unadjusted analysis

^b^ Covariates based on factors associated with HIV acquisition (P<0.10), n = 1372; Due to collinearity, variables partner age difference, partner circumcision status, and history of STIs excluded from the multivariable analysis

^c^ Covariates for stepwise multivariable model based on Akaike information criterion score, n = 1372.

## Discussion

Overall, the simplified risk score performed moderately based on the Brier score at predicting risk of HIV acquisition during pregnancy and postpartum period in our external validation cohort, with an AUC lower than previously reported by Pintye *et al* (0.60, 95% CI: 0.51, 0.69 vs. 0.76, 95% CI: 0.67, 0.85, respectively); indicating poor performance. We found the optimal cut-point for the risk score in the external validation sample was 6.5, the same cut-point identified by Pintye and colleagues; a risk score >6 was associated with a nearly 3-fold increased odds of HIV acquisition, identifying more than a quarter of women as high risk. Half of all women with incident HIV infection would have been correctly classified as high risk. Although risk scores have utility in identifying individuals at risk for HIV acquisition, performance reproducibility of risk score may vary, with the same score demonstrating both poor and moderate predictive ability [1,24,25], highlighting the impact of different settings and populations on risk score performance.

Prioritizing strategies for early detection, such as a simple HIV risk score that can be administered by a healthcare worker in antenatal and postnatal care settings using indicators routinely assessed has the potential to avert incident maternal and pediatric HIV acquisition. While the risk score performance was lower in our external validation than in the initial internal validation, maternal characteristics (having a partner of unknown HIV status and syphilis [17]) included in the simplified risk score are robust and remained predictive of HIV acquisition. Utilizing dual HIV/syphilis testing capable of simultaneously testing for both infections into antenatal care has been shown to be cost effective, and is now recommended by WHO as a first line HIV test [26]. Use of dual HIV/syphilis rapid diagnostic testing offer opportunities to increase coverage of syphilis testing and treatment within MCH settings, and has potential to identify women to prioritize for HIV retesting later during pregnancy or postpartum. In addition, this strategy aligns with WHO guidance and goals for EMTCT of both HIV and syphilis [27].

In the analysis of this cohort, we found that early age at sexual debut was an additional cofactor for HIV acquisition. Prior studies in other populations and settings have found similar results [28,29]. This cofactor could easily be added to the variables routinely collected in the context of MCH clinical settings to identify women at highest risk for HIV and prioritize delivery of prevention interventions, such as PrEP.

Our analysis had several strengths. Few HIV risk assessment tools have been externally validated, and our analysis permitted assessment of utility of implementation and whether performance was altered based on changing epidemiology and prevention interventions. We evaluated additional potential correlates for HIV acquisition, including familiarity with PrEP as an HIV prevention intervention, and ever use of PrEP. While the PrEP variables were not significant in the analysis, complementary efforts to identify high risk women to target with HIV prevention interventions, such as PrEP, may bolster testing and treatment approaches for EMTCT.

Our study has limitations. We only included women in our analysis with complete data on all variables assessed in the risk score; while this may bias our results, multiple imputation may also introduce bias in assessment of risk score performance [30]. Additionally, inclusion of women with incident infection from active case finding may have increased the frequency of HIV in our study, which would bias PPV, NPV, and NNS. The simplified HIV risk assessment tool was originally developed and internally validated using data from a longitudinal study of pregnant and postpartum women; although we externally validated this risk score tool in a population of pregnant and postpartum women from the same region in Kenya, we used data from a cross-sectional study, which would fail to detect changes in risk factors over time. Given the cross-sectional nature of this study, risk factors were assessed concurrently with, rather than prior to, diagnosis of incident infection which may have limited the predictive estimate. Furthermore, the sample size, and number of women with incident infections, for the analysis to explore potential additional risk factors for HIV acquisition was small and led to imprecise estimates of risk.

In summary, using indicators routinely assessed in antenatal care, the external validation of the simplified risk score did not perform as well as it had initially at predicting risk of HIV acquisition. However, in settings with limited resources and in the absence of other tools to ascertain who to prioritize for retesting or PrEP services, this tool may still be valuable. Further evaluation of the simplified risk score in various resource limited settings is needed to determine utility for prioritizing retesting efforts and prevention strategies to avert HIV acquisition among pregnant, postpartum, and breastfeeding women who would most benefit.

## Supporting information

S1 Checklist STROBE statement—checklist of items that should be included in reports of observational studies for manuscript (PONE-D-23-16018). (DOCX)

S1 Table Correlates of HIV acquisition for women receiving maternal and child health care during pregnancy and postpartum in western Kenya. Notes: * among partnered women only in unadjusted analyses; polygamous relationship excluded to allow unmarried women to be included in the multivariable model; ^a^ Unadjusted analysis; ^b^ Covariates based on factors associated with HIV acquisition (P<0.10), n = 2774; Due to collinearity, variables marital status, partner age difference, and syphilis were excluded from the multivariable analysis; ^c^ Covariates for stepwise multivariable model based on Akaike information criterion score, n = 2774.
(DOCX)
