# Supplementary material for: Evaluating the performance of a risk assessment score tool to predict HIV acquisition among pregnant and postpartum women in Kenya
Source: PLoS One. 2024 Jul 10;19(7):e0306992. doi: 10.1371/journal.pone.0306992 (PMC11236202; doi:10.1371/journal.pone.0306992)
Supplement: S1 Table — Notes: * among partnered women only in unadjusted analyses; polygamous relationship excluded to allow unmarried women to be included in the multivariable model; a Unadjusted analysis; b Covariates based on factors associated with HIV acquisition (P<0.10), n = 2774; Due to collinearity, variables marital status, partner age difference, and syphilis were excluded from the multivariable analysis; c Covariates for stepwise multivariable model based on Akaike information criterion score, n = 2774. (DOCX) [file pone.0306992.s002.docx]

| **S1 Table. Correlates of HIV acquisition for women receiving maternal and child health care during pregnancy and postpartum in western Kenya** | | | | | | | | | | | |
| --- | --- | --- | --- | --- | --- | --- | --- | --- | --- | --- | --- |
|  | Univariate^a^ | | |  | Multivariable^b^ | |  | | Stepwise^c^ | | |
| Enrollment characteristics | n^a^ | OR (95% CI) | p-value | aOR (95% CI) | | p-value |  | aOR (95% CI) | | p-value | |
| Demographic characteristics |  |  |  |  | |  |  | | |  |  |
| Age <21 y | 2806 | 0.94 (0.48, 1.83) | 0.86 |  | |  |  | | |  |  |
| Education <8 y | 2802 | 0.77 (0.24, 2.49) | 0.66 |  | |  |  | | |  |  |
| Married | 2765 | 1.94 (0.93, 4.04) | 0.08 |  | |  |  | | |  |  |
| Polygamous relationship* | 1990 | 2.89 (1.25, 6.69) | 0.01 |  | |  |  | | |  |  |
| Relationship duration (in years)* | 2000 | 1.02 (0.94, 1.10) | 0.70 |  | |  |  | | |  |  |
| Partner characteristics |  |  |  |  | |  |  | | |  |  |
| Partner age difference (years older)* | 1883 | 1.07 (1.00, 1.15) | 0.06 |  | |  |  | | |  |  |
| Partner uncircumcised* | 1945 | 0.48 (0.20, 1.15) | 0.10 |  | |  |  | | |  |  |
| Partner HIV status unknown | 2785 | 3.81 (1.93, 7.53) | <0.001 | 3.76 (1.90, 7.44) | | <0.001 | 3.76 (1.90, 7.44) | | | <0.001 | |
| No partner | 2797 | 0.55 (0.25, 1.18) | 0.13 | 1.06 (0.44, 2.58) | | 0.89 | 1.06 (0.44, 2.58) | | | 0.89 | |
| Sexual behavior and practices |  |  |  |  | |  |  | | |  | |
| Age at sexual debut <17 y | 2806 | 1.70 (0.86, 3.35) | 0.13 |  | |  |  | | |  | |
| Age at sexual debut unknown | 2806 | 1.09 (0.43, 2.77) | 0.86 |  | |  |  | | |  | |
| Lifetime no. of sexual partners | 2686 | 0.80 (0.61, 1.06) | 0.12 |  | |  |  | | |  | |
| Any condomless sex in the past month | 2806 | 0.97 (0.87, 1.10) | 0.69 |  | |  |  | | |  | |
| Ever heard of PrEP | 1381 | 0.84 (0.43, 1.64) | 0.60 |  | |  |  | | |  | |
| Ever used PrEP | 1381 | 1.56 (0.47, 5.20) | 0.47 |  | |  |  | | |  | |
| STI and genital tract infections |  |  |  |  | |  |  | | |  |  |
| History of STIs | 2795 | 5.38 (1.60, 18.18) | 0.01 | 4.76 (1.38, 16.39) | | 0.01 | 4.76 (1.38, 16.39) | | | 0.01 |  |
| Syphilis | 1382 | 9.55 (1.96, 46.62) | 0.01 |  | |  |  | | |  |  |
| Notes: * among partnered women only in unadjusted analyses; polygamous relationship excluded to allow unmarried women to be included in the multivariable model; ^a^ Unadjusted analysis; ^b^ Covariates based on factors associated with HIV acquisition (P<0.10), n=2774; Due to collinearity, variables marital status, partner age difference, and syphilis were excluded from the multivariable analysis; ^c^ Covariates for stepwise multivariable model based on Akaike information criterion score, n=2774 | | | | | | | | | | | |
